# Physical Activity Readiness in Patients with Heart Failure

**DOI:** 10.3390/ijerph192316332

**Published:** 2022-12-06

**Authors:** Elena Marques-Sule, Pallav Deka, Luis Almenar, Dola Pathak, Raquel López-Vilella, Leonie Klompstra

**Affiliations:** 1 Physiotherapy in Motion, Multispeciality Research Group (PTinMOTION), Department of Physiotherapy, University of Valencia, 46010 Valencia, Spain; 2 Unidad de Insuficiencia Cardíaca y Trasplante, Servicio de Cardiología, Hospital Universitari i Politècnic La Fe, 46026 Valencia, Spain; 3 College of Nursing, Michigan State University, East Lansing, MI 48824, USA; 4 Department of Medicine, University of Valencia, 46010 Valencia, Spain; 5 Centro de Investigación Biomédica en Red de Enfermedades Cardiovasculares (CIBERCV), Instituto de Salud Carlos III, 28029 Madrid, Spain; 6 Department of Statistics and Probability, Michigan State University, East Lansing, MI 48824, USA; 7 Department of Health, Medicine and Caring Sciences, Linkoping University, 58183 Linkoping, Sweden

**Keywords:** physical activity, physical readiness, motivation, self-efficacy, exercise, heart failure

## Abstract

The aim of this study was to explore the readiness for physical activity (PA) and its related factors in patients with heart failure. This cross-sectional study included 163 patients with heart failure (mean age 66 ± 16, 50% female). The ability to safely engage in PA was assessed with the PA Readiness Questionnaire (PAR-Q). Psychological readiness was measured using two questionnaires, namely: Exercise Self-efficacy Scale and the Motivation for PA and Exercise/Working Out. A multivariate analysis of covariance was conducted to test the effect of background variables on readiness for PA. 64% (n = 105) of patients reported not being able to safely engage in PA, 80% (n = 129) reported low self-efficacy, and 45% (n = 74) were extrinsically motivated indicating external factors drove their motivation. Factors that positively influenced the PA readiness included lower age (*p* < 0.01), being male (*p* < 0.01), being married (*p* < 0.01), having higher education (*p* < 0.01), being in NYHA-class I compared with II (*p* < 0.01), less time since diagnosis (*p* < 0.01), lower BMI (*p* = 0.02), and not suffering from COPD (*p* = 0.02). Prior to recommending exercise, assessment of safety to engage in PA along with self-efficacy and motivation in patients with heart failure is essential.

## 1. Introduction

Heart failure (HF) is a chronic syndrome with a prevalence and incidence on the rise due to an ageing population and increased survival from ischemic and non-ischemic causes [1,2]. An estimated 64 million people are living with HF worldwide [3], with a prevalence estimated at 1% to 2% of the general adult population in developed countries [4,5,6]. The economic burden of HF is high, especially from costs associated with hospitalization and frequent readmissions. Reducing admission rates in patients with HF is the most promising approach to decrease the economic burden of HF [7].

Self-management strategies, such as physical activity (PA, any bodily movement produced by skeletal muscles that results in energy expenditure [8]), can optimize health outcomes and decrease hospitalization in patients with heart failure [1,2,9]. Despite these benefits, a large proportion of patients with HF are not engaging in PA regularly [2,9,10].

Education is an essential component of nearly all management programs for patients with HF. Although education typically addresses the changes in PA necessary to control signs and symptoms and improve outcomes, failure to adhere to recommendations continues to be a common reason for hospital readmission for patients with HF [2,10]. Education alone does not guarantee changes in PA as readiness for PA and exercise is essential for behavioral changes. Exercise is a subset of PA; it is structured and repetitive in nature and is performed with the intention of improving physical fitness [8]. Little attention has been given to assessing the readiness, motivation, and self-efficacy of patients with HF to make lifestyle and behavioral changes [2,9]. Readiness is defined as the “state of preparation” [11]. As such, it is a state as well as a process for taking action [12]. When exploring PA as a self-management approach for patients with HF, it is important to consider the concept of readiness as it relates to health behavior change. Readiness can be understood as an individual’s predisposition to engage in a health behavior change or an indication of a central motivating force [13]. As such, readiness for PA not only has a physical component (the ability to safely engage in activity that involves movement) but is also intricately connected to the individual’s motivation and self-efficacy for the activity. According to the Self-Determination Theory (SDT), motivation for performing an activity can be intrinsic (sheer pleasure in performing the task) or extrinsic, which is further classified into external regulation (doing it just to get a reward or avoiding punishment), introjected regulation (to gain approval or avoid disapproval), identified regulation (the activity has been somewhat internalized as a consciously valued goal), or integrated regulation (activity’s value has been fully internalized and integrated) [14]. The literature provides good evidence for the value of SDT in understanding exercise behavior, demonstrating the importance of autonomous (integrated and intrinsic) regulations in fostering PA. Whereas identified regulation predicts initial/short-term adoption more strongly than introjected motivation, integrated extrinsic motivation or intrinsic motivation is more predictive of long-term exercise adherence [15].

Self-efficacy, according to the Social Cognitive Theory, refers to an individual’s confidence in his or her ability to engage in a behavior, even when barriers occur [16]. A higher self-efficacy is associated with greater PA participation in cardiac patients [17,18,19]. The ability to safely engage in PA, motivation, and self-efficacy may be the underlying factors for high drop-out rates and why interventions to improve PA and exercise do not achieve desired outcomes in this population [20,21].

Readiness in PA, as a combination of the ability to safely engage in PA (based on health history, current symptoms, and risk factors) and psychological readiness to engage in PA (individual’s motivation and self-efficacy for the activity), has not been previously explored in patients with HF. Therefore, the primary purpose of this study is to explore the readiness for PA in patients. The secondary aims were to assess daily PA in patients with HF and its association with readiness for PA. Additionally, we explored factors associated with readiness for PA in this population.

## 2. Materials and Methods

### 2.1. Design and Participants

This is a cross-sectional study. Patients diagnosed with HF (regardless of their ejection fraction), free from major psychiatric or neurocognitive conditions, and older than 18 years of age were eligible for participation. Exclusion criteria were inability to understand Spanish and/or cognitive impairments that would make it impossible to fill in the study questionnaires.

### 2.2. Procedures

The present study complies with the Declaration of Helsinki and was approved by the Regional Ethics Committee (H1510342565432).

All patients with HF that visited the cardiology department in a university hospital in Spain (Valencia) between January–July 2018 were invited by research staff to participate. Patients provided oral and written informed consent before filling out the questionnaires. Help was provided for filling in the questionnaire when needed.

### 2.3. Measurements

Demographic and clinical characteristics including age, gender, time since diagnosis, New York Heart Association Classification (NYHA-class), body mass index (BMI), and comorbidities (diabetes, chronic obstructive pulmonary disease (COPD)) were collected from the patients’ medical charts. Information on marital status and educational level was assessed using a questionnaire. 

Readiness for PA was assessed with: (i) the ability to safely engage in PA; and (ii) psychological readiness in becoming more physically active.

The ability to safely engage in PA was assessed with the PA Readiness Questionnaire (PAR-Q) [22]. This questionnaire contains 7 questions and is mostly used as a screening questionnaire to detect participants at risk of adverse events before beginning PA and exercise [16]. Individuals answering yes to any of the questions are advised to consult with their doctor before performing any PA. The weight of the total score of the PAR-Q scale was reversed for analytic purposes and higher scores on the PAR-Q test indicated lower ability to safely engage in PA. This was done to reflect the positive association between PAR-Q and PA.

Psychological readiness in becoming more physically active was measured with the Exercise Self-efficacy Scale (ESES) and motivation with the Motivation for PA and Exercise/ Working Out questionnaire (RM 4–FM). The ESES included 9 items and assessed the participant’s confidence to perform exercise under different conditions or constraints. For each condition or constraint such as being tired, weather conditions, afraid of being hurt, lack of motivation, etc., participants responded on a 7-point scale, ranging from “very sure” (1) to “not at all sure” (4). A score of 15 and higher was seen as having exercise self-efficacy. This scale has been tested for its validity and reliability [23]. The RM 4–FM questionnaire included 16 items and was designed to assess whether the participants are extrinsically or intrinsically motivated to engage in PA. The questionnaire makes a statement of “I try, or would like to try, to be physically active regularly” and the participants have to rate themselves on a Likert scale between 1 (not true at all) to 7 (very true). A mean from each subscale of External Regulation, Introjected Regulation, Identified Regulation, and Intrinsic Motivation was calculated. A mean score of 5 or higher was seen in this study as experiencing this subpart of motivation. Relative autonomy is calculated by the sum of each subscale multiplied by its weight: (Ext Reg × −2) + (Intro Reg × −1) + (Ident Reg × 1) + (Intrins Motiv × 2). A negative score is more related to external motivation, while positive scores relate to internal factors. This scale is valid and reliable [24].

The amount of physical activity was measured with the short form International Physical Activity Questionnaire (s-IPAQ)). The s-IPAQ contains seven items for identifying the frequency and duration of light, moderate, and vigorous PA, as well as inactivity during the past week. The answers to the questions were transformed into Metabolic Equivalent of Task (MET-minutes), a physiological measure expressing the energy cost of physical activities. The patients were classified into three PA categories: low, moderate, and high. The s-IPAQ is a valid and reliable instrument to measure PA [25,26].

### 2.4. Statistical Analysis

In the descriptive analyses, means and standard deviations were calculated for continuous data, and absolute numbers and percentages were computed for nominal variables. The normal distribution of the data was tested by the Kolmogorov–Smirnov test. Correlational analyses were done to explore the relationship between the ability to safely engage in PA and psychological readiness and the total amount of MET-minutes. A multivariate analysis of covariance (MANCOVA) was done to test the effect of categorical variables on PA readiness. Factors that were tested in the model were age, gender, time after diagnosis (months), body mass index (BMI), marital status (married/in a relationship or single), educational level (primary school, secondary school, or university), NYHA-class, and the presence of diabetes and COPD. A *p*-value < 0.01 was considered significant. The basic assumptions to fit the linear model were met. Data were analyzed using SPSS (version 25).

## 3. Results

In total, 163 patients participated (response rate 82%), with 35 % (n = 57) reporting low PA level (<600 MET-minutes a week) and 65% (n = 106) reporting moderate PA level (between 600–3000 MET-minutes a week). Participants had a mean age of 66 (±16) years, 50% were female, 82% were married, 63% NYHA I, and 37% NYHA II (Table 1).

### 3.1. The Ability to Safely Engage in PA and Psychological Readiness

Of the patients included, all patients were taking medication for their heart condition. In our analysis, when this question was discarded, 64% reported not being able to safely engage in PA (n = 105); 43% (n = 70) experienced balance problems, 34% (n = 55) experienced chest pain, and 20% (n = 32) experienced problems in their joints.

Eighty percent of the patients reported low self-efficacy for exercise (n = 129). In total, 45% (n = 74) were extrinsically motivated for PA and 55% (n = 89) reported primarily internal motivating factors to be physically active.

Most components (the ability to safely engage in PA and psychological readiness) of PA readiness were correlated with each other (Table 2); only introjected regulation (r = 0.134, *p* = 0.09) and identified regulation (r = 0.07, *p* = 0.38) were not correlated.

Higher ability to safely engage in PA, higher motivation, and higher self-efficacy were positively associated with the amount of physical activity that patients with HF performed.

### 3.2. Factors Related to Physical Activity Readiness

There was a statistically significant higher PA readiness based on lower age (*p* < 0.01), male gender (*p* < 0.01), less time since diagnosis (*p* < 0.01), higher educational level (*p* < 0.01), being married (*p* < 0.01), and being in NYHA-class I compared with NYHA-class II (*p* < 0.01), lower BMI (*p*-value = 0.018), and not having COPD (0.022). Having diabetes did not influence the PA readiness (*p*-value = 0.963) (Table 3).

## 4. Discussion

A prudent and important first step in the fitness assessments and exercise prescription process in patients with HF is the determination of PA readiness. Along with screening for the ability to safely engage in PA, it is also important to screen for psychological readiness for engaging in PA. Higher ability to safely engage in PA and higher psychological readiness were found to be associated with higher PA in patients with HF.

In our study, not taking medication use in consideration as all patients were on HF medication, more than half of the patients (64%) experienced the inability to safely engage in PA. Commonly reported physical limitations included the experience of balance problems (43%), chest pain (34%), and joint problems (20%). Our findings agree with prior studies. A previous study [27] on patients with HF reported abnormality in balance in 58% of the patients and 25 % reported musculoskeletal pain in the back (29%), knee (17%), and hip (8%).

In analyzing psychological readiness, we found that half of the participants were extrinsically motivated for PA and exercise and the other half experienced internal factors that motivated them to be involved in their PA. Although the strength of the motivation was not strong (mean score of 0.30), this indicates that while one-half of the patients were driven to perform PA for the sheer pleasure that was derived from engaging in PA, the other half was motivated for extrinsic reasons, such as valuing the importance of PA for their health condition, to impress others, attaining a reward, or for fear of punishment. Motivations towards exercise are important in patients with HF, as self-determined patients are more likely to maintain exercise behavior [28]. It is important to increase self-determination motivation; besides increasing PA, this also improves well-being during cardiac rehabilitation and longer-term PA [29]. Using the SDT framework, it can be inferred that participants who are intrinsically motivated to perform PA and exercise are likely to also adhere to the recommendations in the long term. Our study showed that both extrinsic motivational factors (especially identified and integrated regulators) and intrinsic motivational factors are important in PA engagement. Though social motivation was not measured in the current project, previous work [18] has highlighted that social motivation was expressed as the least important motivation to be physically active (22%), physical motivation was expressed to be important by 33%, and psychological motivation was rated as the most frequent motivation for being physically active (41%) [18]. As such, a one-size-fits-all strategy to increase motivation will not address the specific needs of patients with HF. It is, therefore, important to assess what motivates patients to be physically active and offer individualized strategies (e.g., goal setting, motivational counseling, or interviewing) for better integration of PA and exercise in their lifestyle.

Motivation to be physically active is important but not sufficient. In addition to a high level of motivation to be physically active, it is important that patients with HF have a high degree of self-efficacy [19]. Patients with higher self-efficacy were likely to spend more energy by being physically active. In our sample, most patients (80%) experienced low self-efficacy, which was also reflected in participants engaging in low-moderate levels of PA. It is also a predictor for adherence in cardiac rehabilitation programs [18,30]. Self-efficacy can change over time and recalibration of self-efficacy with exposure to PA has been demonstrated [31]. With HF patients tending to experience peaks and troughs in their health status with disease progression, it becomes imperative to frequently assess self-efficacy for PA for designing optimal strategies for enhancing efficacy. A previous study [32] found that processes of change and self-efficacy were found as the most important predictors of a patient’s readiness to act. This indicates that strategies to increase self-efficacy should be adapted to the patient’s stage of behavioral change. Strategies for improving self-efficacy that have been shown to improve PA adherence, including help with mastery of the skill, applying vicarious experience, and providing feedback and persuasion for PA, should be utilized by future clinical trials [31].

Our study shows that the ability to safely engage in PA and psychological readiness are highly correlated with each other, meaning that they influence each other, confirming our study’s outcome measure of PA readiness. We found the inability to safely engage in PA and psychological readiness among the participants in our study. Our conclusion is supported by other studies [33,34] that found half of cardiac patients had no intention to become more physically active, and although patients believe that they were doing well in controlling their risk factors, a limited number of patients performed PA to control these risk factors.

Factors that positively influenced the PA readiness included lower age, being male, being married, having higher education, lower NYHA classification, less time since diagnosis, lower BMI, and not suffering from COPD. Other studies confirm our outcomes. One study showed that women experienced more physical, social, and psychological motivations toward physical activity compared with men. Women often express being proud of themselves when they are regularly physically active and attribute their success to being in good physical shape. The HF-ACTION study [35] showed that COPD in patients with HF was associated with older age, more comorbidities, reduced exercise capacity, and increased CV mortality/HF hospitalization. This study also showed that there was no differential response to exercise training in patients with HF and COPD compared with patients with HF. Previous studies show that the presence of other comorbidities was also associated with low participation in cardiac rehabilitation and exercise [36,37]. In an interview study [38], patients with HF expressed that they normalized a sedentary lifestyle as part of getting older, and that they felt restricted by co-morbidities and focused on what they could no longer do because of HF. This study, involving NYHA Class II, III, and IV HF patients, also showed that experiencing symptoms during physical activity negatively influenced PA. It is important to recognize that physical activity should be tailored to the specific needs of patients with HF and COPD. Patients who have HF and COPD should be followed-up by multidisciplinary and multi-professional healthcare teams devoted to the management of patients with chronic-degenerative diseases.

Obesity is a risk factor for inactivity and our study found that obesity is also associated with low PA readiness. A previous study [39] showed that compared with the non-obese elderly, the moderately obese had two times the risk of inactivity and the severely obese had four times the risk of inactivity. This study also showed that poor health, pain, diseases, and tiredness explained 27% of the increased risk of physical inactivity. Fear of falling and injury and being uncomfortable and insecure when performing PA contributed 23% to the increased risk of inactivity. Another study [40] found that BMI and aerobic capacity were significantly associated with physical activity readiness.

The findings of this study can assist nurses and other healthcare providers worldwide by highlighting the need for screening for PA readiness in HF patients to reduce the risk associated with engagement in PA and improving adherence to exercise guidelines. There are some limitations in this study. The cross-sectional design was a limitation in implying causal relationships between PA readiness. The generalizability of these findings may be limited to relatively healthy HF patients; therefore, the disease itself could be of little influence on the PA readiness. The population was also younger than the general HF population. This could be because the research included relatively healthy HF patients, as patients included were classified as NYHA I or II, with comparatively higher levels of functional ability as compared with NYHA Class III and IV patients with HF. Therefore, generalization of the results of this study to the entire HF population is cautioned. In addition, the focus of this study is predominantly on short-term exercise uptake, although the importance of long-term physical activity adherence should be highlighted in this regard. We use the PAR-Q for assessing the ability to safely engage in PA. We recommend the use of the PAR-Q+ to assess physical readiness in future studies as it may be more sensitive in detecting safety issues in engaging in PA [41]. Another limitation was that we tested limited factors that could have contributed to PA readiness, such as comorbidities, medication use, and dyspnea.

## 5. Conclusions

This study shows that PA readiness has both a physical and a psychological component. More than half of the patients were not able to engage safely in PA. Motivation (both extrinsic motivation and internal factors that motivate) was found to be important in PA, but most patients experienced low self-efficacy towards PA. Factors that positively influenced PA readiness included lower age, being male, being married, having higher education, lower NYHA classification, less time since diagnosis, lower BMI, and not suffering from COPD. When prescribing PA prescriptions to patients with HF, we should not only assess if it is safe for them to engage in PA but also assess if they are motivated and have self-efficacy to be able to become more physically active.

## Figures and Tables

**Table 1:** Demographic and clinical characteristics of 163 patients with heart failure.

	n = 163
**Age (years) (mean ± SD)**	66 ± 16
**Male gender (% n)**	50% (81)
**Time since diagnosis (months) (median, IQR)**	60 (24–120)
**Educational level (% n)**	
Primary school	62% (101)
Secondary school	23% (38)
University	15% (24)
**Marital status (% n)**	
Married	82% (134)
Single	18% (29)
**NYHA (% n)**	
I	63% (102)
II	37% (61)
**BMI (mean ± SD)**	27 ± 3
Normal (18.5–24.9) (% n)	26% (43)
Overweight (25–29.9) (% n)	58% (94)
Obese (30 or higher) (% n)	16% (26)
**Comorbidities (% n)**	
Diabetes	35% (57)
COPD	7% (12)

BMI, body mass index; COPD, chronic obstructive pulmonary disease; NYHA, New York Heart Association Classification.

**Table 2:** Correlations between physical readiness and psychological readiness.

			Ability to Safely Engage in Physical Activity	Psychological Readiness					Physical Activity
				Motivation					
				External Regulation	Introjected Regulation	Identified Regulation	Intrinsic Motivation	Self-Efficacy	
**Ability to safely engage in physical activity**			-	0.371 **	0.377 **	0.378 **	0.666 **	0.580 **	0.301 **
**Psychological readiness**	Motivation	External regulation	0.246 **	-	0.689 **	0.644 **	0.566 **	0.219 **	0.249 **
		Introjected regulation	0.134	0.689 **	-	0.569 **	0.535 **	0.331 **	0.332 **
		Identified regulation	0.069	0.644 **	0.569 **	-	0.679 **	0.183 *	0.268 **
		Intrinsic motivation	0.258 **	0.566 **	0.535 **	0.697 **	-	0.347 **	0.410 *
	Self-efficacy		0.241 **	0.219 **	0.331 **	0.183 *	0.347 **	-	0.274 **

* *p*-value < 0.05 ** *p*-value < 0.01.

**Table 3:** Multivariate general linear model with physical activity readiness (physical and psychological readiness) as dependent variables.

	Wilk’s Λ	F	*p*-Value
Age	0.714	8.414	<0.001
Gender	0.837	4.080	0.001
Time since diagnose	0.837	2.812	0.009
Educational level	0.678	9.994	<0.001
Marital status	0.767	6.373	<0.001
NYHA	0.832	4.241	<0.001
BMI	0.893	2.512	0.018
Diabetes	0.987	0.275	0.963
COPD	0.897	2.420	0.022

COPD: chronic obstructive pulmonary disease; NYHA: New York Heart Association Classification.

## Data Availability

The data that support the findings of this study are available from the corresponding author upon reasonable request.
